# Turning Neural Prosthetics Into Viable Products

**DOI:** 10.3389/frobt.2021.754114

**Published:** 2021-09-29

**Authors:** Gerald E. Loeb, Frances J. Richmond

**Affiliations:** ^1^ Medical Device Development Facility, Department of Biomedical Engineering, University of Southern California, Los Angeles, CA, United States; ^2^ DK Kim International Center for Regulatory Science, Department of Regulatory and Quality Sciences, University of Southern California, Los Angeles, CA, United States

**Keywords:** regulation, reimbursement, intellectual property, risk management, premarket approval, return on investment

## Abstract

Academic researchers concentrate on the scientific and technological feasibility of novel treatments. Investors and commercial partners, however, understand that success depends even more on strategies for regulatory approval, reimbursement, marketing, intellectual property protection and risk management. These considerations are critical for technologically complex and highly invasive treatments that entail substantial costs and risks in small and heterogeneous patient populations. Most implanted neural prosthetic devices for novel applications will be in FDA Device Class III, for which guidance documents have been issued recently. Less invasive devices may be eligible for the recently simplified “*de novo*” submission routes. We discuss typical timelines and strategies for integrating the regulatory path with approval for reimbursement, securing intellectual property and funding the enterprise, particularly as they might apply to implantable brain-computer interfaces for sensorimotor disabilities that do not yet have a track record of approved products.

## Overview

**FIGURE 1:**
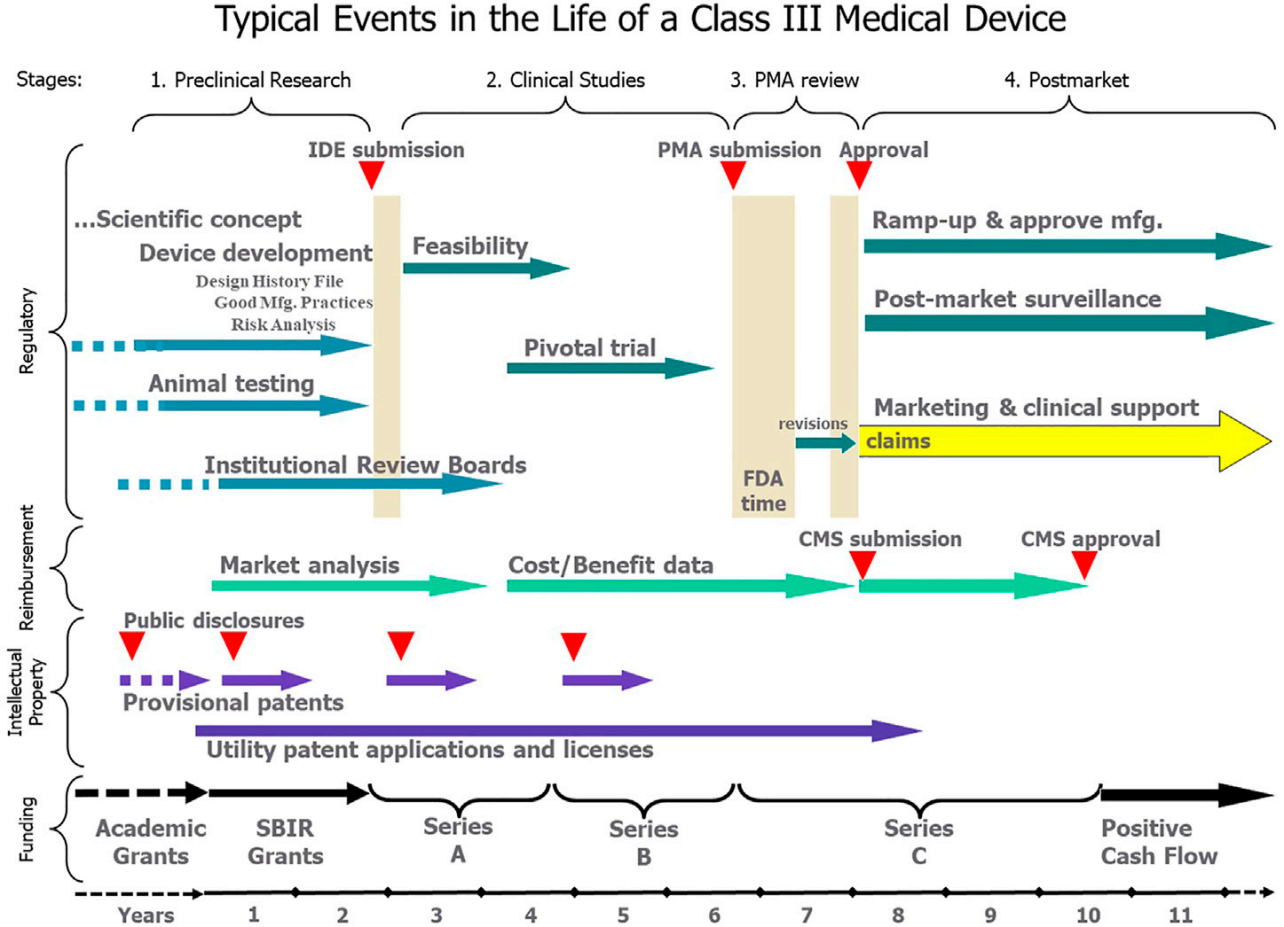
After an indeterminate number of years of funding and development as an academic project (dashed lines), the typical transition to a commercial product can be divided into four stages, each of which involves teams of non-technical personnel for Regulatory, Reimbursement, Intellectual Property and Funding activities over a period of years. All these activities should be focused on the approved claims that eventually provide the basis for marketing and revenue from sales of an approved, reimbursed and protected product.

Academic researchers who develop a novel technology will want it used to treat life-altering sensorimotor dysfunction. In the capitalist societies of most industrialized nations, this means building a commercially successful business. However, such aspirations involve activities and expertise far beyond the skillset of most academic researchers. We outline those processes here so that academic founders of such projects will understand the scope of the undertaking and recruit the necessary expertise over the product life cycle (Figure 1).

The FDA recognizes the growing translational research and clinical promise of such devices and has published recent guidance documents that pull together the regulatory requirements likely to pertain to sensorimotor prosthetic systems [see Implanted Brain-Computer Interface (BCI) Devices for Patients with Paralysis or Amputation–Non-clinical Testing and Clinical Considerations, https://www.fda.gov/media/120362/download]. Such systems usually include implanted components plus external controllers and interfaces to be operated by patients and clinicians. Their subsystems usually contain extensive custom software, each element of which has its own risk profile and requirements for testing. The regulatory requirements related to the complete risk profile dominate the considerations for how to manage a commercializable product. The Medical Device Amendments of 1976 require that medical devices be classified according to risk, and higher risk products follow a rigorous development and testing process that culminates in some form of submission to the US Food and Drug Administration (FDA). These rules are particularly stringent for novel and invasive technologies whose unknown risks and uncertain safety and efficacy profiles typically place them in the highest Class III. This article is focused on such Class III devices.

We have organized this paper according to the typical chronology of technology transfer from academic research to successful product (Figure 1). We discuss when and how the arduous but well-described Class III regulatory path interacts with strategies for marketing and reimbursement, intellectual property management and corporate funding.

Experience has shown that commercial success and clinical availability can depend at least as much on business considerations as on the underlying science and technology of the product (Brown et al., 2008). For example, the Clarion® multichannel cochlear implant was derived from a forerunner developed at UCSF and tested in a small number of patients under NIH grants and contracts from about 1974–1983 (Loeb et al., 1983). An initial transfer to a manufacturer was unsuccessful 1983–1987. The project was taken over by the Alfred Mann Research Foundation and thoroughly reengineered for manufacture by Advanced Bionics Corp., a spin-off company ∼1990. FDA PreMarket Approval as a Class III medical device for adults was obtained in 1995. Reimbursement by insurance companies was a long battle and the product was not profitable until ∼1999, at which time the company was sold initially to Boston Scientific and eventually to Sonova Holding AG. The initial market was adults with postlinguistic profound deafness, a small population. Successful results eventually led to expansion to much larger markets including severely deaf adults and prelinguistically deaf children. The Clarion has two major international competitors with similar market shares, all derived from academic projects ∼40 years ago. Another ∼20 academic projects in this field never resulted in successful products (Loeb, 1990). This extended time course is probably not unusual when pioneering a new neural prosthetic technology.

## Stage 1: Preclinical Research

Neural prosthetic interfaces are built on decades of fundamental research in neurophysiology, clinical pathology and technology that have typically been conducted in academic institutions and funded by grants from government agencies. Such “concept phase” research is subject to local regulations regarding animal care and human subject protection but is not considered part of the “regulated” activities to develop a commercial product. The decision to pursue a clinical product requires a major change in thinking around every aspect of the research. The original funding agencies needed to be convinced of a general clinical need, but investors need to see viable markets. Small markets may offer less expensive regulatory and marketing paths but fewer opportunities to recoup investment, as discussed below. Market analysis should therefore include the current and projected incidence of the underlying disorder, the prevalence of patients with disabilities severe enough to warrant the proposed treatment, comorbidities that might contraindicate such treatment, and the current and projected availability of competing solutions (Loeb, 2018). The first activities in this transitional phase require decisions about the intended market, the regulatory path and the highly disciplined developmental activities that can produce the well-specified and well-tested final product ready for formal clinical trials.

### Regulatory Considerations

Regulatory decisions must be made very early because they set some of the design and testing requirements for market approvals in different countries. Typically, product developers will try to understand the fit of their product in the classification systems of the US and/or the EU, the two largest markets. These approaches usually produce the kinds of data needed for product registration in other countries (Kedwani et al., 2019). In the early stages the developmental activities that they call out as required elements are relatively similar across countries because the complex and risky nature of the new systems place them in the highest-risk categories. In the US, the highest classification, Class III, includes risky implantable systems, such as deep brain stimulators for Parkinson’s disease, dystonias and epilepsy, and novel devices that have yet to be classified. All Class III products must follow the rules and steps leading to eventual submission of a Premarket Approval Application (PMA) or a modified Humanitarian Device Exemption for products whose applications address rare conditions, as described below. The first stage of regulatory interaction for such products is typically the submission of an Investigational Device Exemption (IDE) to use devices experimentally in humans in order to collect data on safety, efficacy and performance.

New or modified medical devices that are substantially equivalent to a predicate device in Class I or II in terms of indications, claims and risks are eligible for the 510 (k) approval route, which often does not require clinical data. Novel medical devices lacking such predicates are in Class III, but the manufacturer of a low-risk device can pursue a De Novo Classification that will both permit commercialization and assure that the product is classified in the lower Class I or II categories going forward. Previously, De Novo applications were allowed only after a failed 510 (k), but this was recently amended to permit the immediate pursuit of a De Novo classification when no legally marketed predicate exists (https://www.fda.gov/medical-devices/premarket-submissions/de-novo-classification-request).

Novel neural prostheses aimed at relatively small markets such as quadriplegia are often eligible for FDA’s Humanitarian Use Designation (HUD). As of 2016, this category includes any medical device intended to benefit patients in the treatment or diagnosis of a disease or condition that affects or is manifested in not more than 8,000 individuals in the United States per year (https://www.congress.gov/114/bills/hr34/BILLS-114hr34eah.pdf). Additionally, the device might qualify if the targeted disease has a larger population but there is an “orphan subset” that has a special need for the device. Qualification as an HUD allows the company to replace the PMA submission with a submission for Humanitarian Device Exemption (HDE) in which data from a much smaller clinical trial can be used to demonstrate safety and “probable efficacy.” HDE approval carries strict limitations on sites of use, labeling, marketing and pricing (https://www.meddeviceonline.com/doc/does-the-humanitarian-device-exemption-process-work-and-is-it-worth-pursuing-0001). Because most products rely on larger markets for commercial success, companies will typically plan to extend the original clinical trials to obtain efficacy data in support of a full PMA (Bernad, 2009). For this purpose, data collected from HDE sales can be helpful.

In the earliest stages of development, researchers often take advantage of the FDA Custom Device Exemption (https://www.fda.gov/media/89897/download). If the device is not generally available and is designed or modified to meet the needs of an individual patient, the FDA waives IDE requirements for up to five patients per year (Klepinski, 2006). It is then up to the prescribing physician, the local institutional review board (IRB), the manufacturer and the hospital to assume the responsibility (and liability) for the care of each patient. The usage must be for the benefit of the patient rather than to collect data for a regulatory submission. However, published case studies can provide valuable “real world evidence” to advance the technology and its eventual clinical acceptance.

In the EU, it is likely that most implantable neural prostheses will be classified in Class III for products that are “in direct contact with heart or central circulatory/nervous system.” These products will obtain a CE mark prior to commercialization by working with a private, government-regulated Notified Body that will assure that the new product meets the requirements spelled out in the European Medical Device Regulations and associated standards.

**FIGURE 2:**
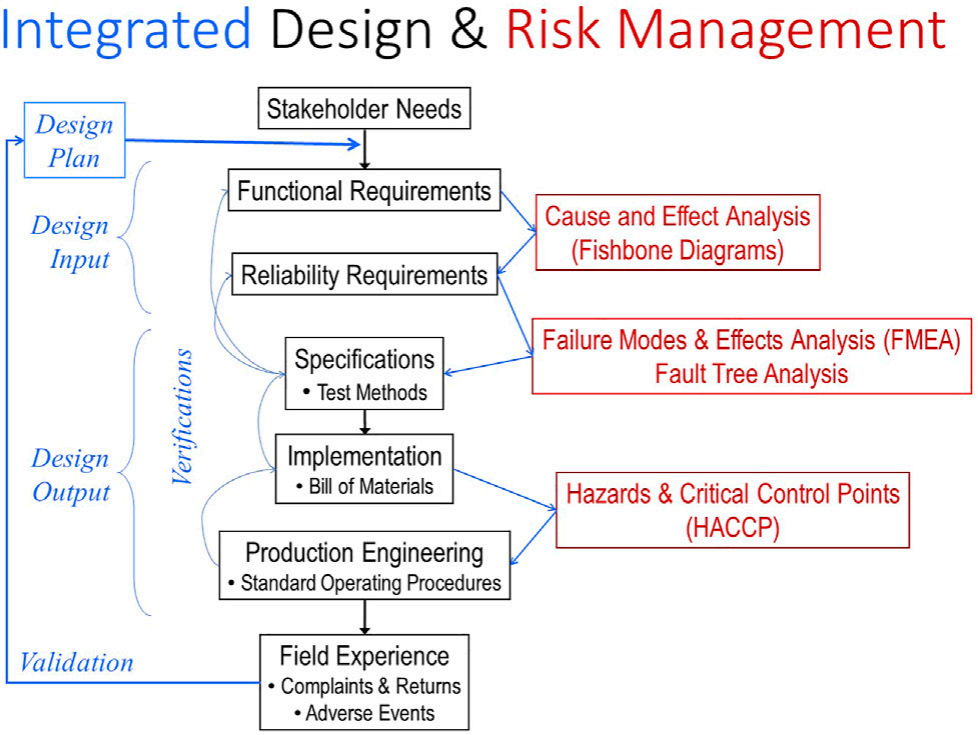
The steps in the FDA-mandated Design Control process (center section in black) must be informed by various Risk Management tools (right section in red) and integrated, verified and documented (left section in blue). When unanticipated complaints and failures occur in the field, they often mandate expensive and lengthy recalls and remediation of the product. They also represent a failure to validate the design control and quality processes, which should then be modified to prevent similar failures with future products.

Regulatory submissions for Class II or III devices require a well-developed technical file based on a set of required steps and documentation called Design Controls in the US (21 CFR 820.30 and 820.40, respectively) (central portion of Figure 2). These were put in place in the 1990s when it became apparent that about half of adverse events were caused by medical devices that conformed to their manufacturing specifications but were designed badly for their actual use. The basic requirement is to start with a complete capture of the needs of all stakeholders in a set of functional requirements against which subsequent steps of product specification and implementation can be verified systematically (Loeb and Richmond, 2003). The goal is to avoid unanticipated adverse events or other failures when products are in normal use by clinicians, patients and other stakeholders. Most Class III medical devices are manufactured by mature companies that have spent many years refining their design processes through the experience of such validation (left portion of Figure 2). Part of this evaluation can involve the clinical trials themselves, but human factors studies to anticipate use errors or ergonomic concerns are also required by regulatory agencies (Privitera et al., 2017).

Over the last 20 years Design Controls have become integrated with tools for Risk Management, a mandated and critical process for identifying all hazards that might be presented by a medical device system (right portion of Figure 2). These tools allow quantification of probability and severity of failures of components, systems and manufacturing processes, estimates of system reliability, and documentation of effectiveness of measures to mitigate such risks. The FDA relies heavily on these analyses by the sponsor when considering novel and complex devices and systems.

Demonstrating the existence of and adherence to Design Controls is mostly foreign to academic research laboratories and personnel. Data that are collected from prototype devices and animal experiments will generally not be accepted in future regulatory submissions unless the devices have been produced under Quality Systems and tested according to Good Laboratory Practices, which require specialized approaches and personnel that usually cannot be funded by research grants. Software validation, sterilization methods, and shipping and storage requirements are often overlooked and can threaten the success of a design, so must be integrated early into the design and testing strategy. Thus, new companies started by researchers often find it necessary to work with or be acquired by a larger commercial enterprise as they approach late-stage preclinical or early clinical product development.

### Reimbursement Considerations

The IDE/PMA regulatory path is concerned primarily with identifying and supporting the claims of safety and efficacy for approved medical indications. Such claims limit the size of the market and the value of the product, so they are a key determinant of its commercial viability. Potential markets should be assessed according to incidence and prevalence of all possible indications, the medical and financial consequences of currently available and potentially emergent treatments, and the likelihood of demonstrating statistically significant benefits that outweigh risks and costs. These considerations inform the design of the product that will address them successfully, a concept known as target product profile (TPP). Wise selection of promising markets and the claims required to succeed in them is essential for the design of efficient and successful clinical trials in the next phase.

### Intellectual Property Considerations

Many of the inventive aspects of the technology and its clinical application are likely to emerge during the academic life of the project, when faculty and students are under pressure to publish their research in a timely manner. Such public disclosure means that any subsequent patent application will not be considered as novel, thereby eliminating most or all opportunities to obtain intellectual property protection for such inventions. Most investors will be unlikely to commit the large sums required to commercialize a medical product without patent protection against cheaper imitations (Ma, 2015).

The US Patent and Trademark Office provides a rapid and inexpensive way to secure such inventions by filing a provisional patent application, thereby securing a priority date for the invention before any public disclosure. If the research was funded by the US government, it falls under the provisions of the 1980 Bayh-Dole Act (https://en.wikipedia.org/wiki/Bayh-Dole_Act), which grants ownership of and agency to pursue patents to the grantee institution. The inventors should disclose their invention to their institution’s technology transfer office, which can submit whatever technical description is available as a provisional patent application for a $280 filing fee. The technical information will not be reviewed by an examiner, but the inventors then have 1 year to file a complete utility patent application before they lose their priority date (https://www.uspto.gov/patents/basics/types-patent-applications/provisional-application-patent).

If the project is transferred to a commercial entity during this phase, then it is essential for that entity to receive a license for the intellectual property. If the property has not yet been secured by an issued US patent, then the entity will probably desire or be required to take over the prosecution of any pending provisional or utility patents. This process is typically lengthy (3–5 years) and expensive ($10–25 K for even simple inventions in the US alone; https://www.bitlaw.com/guidance/patent/what-does-a-patent-application-cost.html). Further inventions are likely during this seminal phase, so ownership and prosecution rights should be clearly established, especially if the commercial entity continues to work collaboratively with an academic institution.

### Funding Mechanisms

Considering the many non-academic activities that should be started in the preclinical phase, it is highly desirable to transfer the project to a commercial entity as early as possible. An established company with experience in Class III devices would be ideal but they are often not willing to tackle truly innovative and high-risk technologies with uncertain markets at this early stage. Further development often falls to start-up companies whose principals are drawn from the founding scientists, engineers and clinicians. Obtaining investors at this early, high-risk stage is difficult and likely to require that founders sell a controlling interest in the company. The federal agencies that fund academic research have congressionally mandated budget set-asides to provide grants to such small businesses, thereby avoiding dilution of ownership. These are the Small Business Innovative Research (SBIR) and Small Business Technology Transfer (STTR) programs, which also facilitate continuing partnerships with academia (National Research Council, 2009; Sun et al., 2021). Unfortunately, such grant funds cannot be used for patent applications, which tend to be a significant expense in this first phase.

## Stage 2: Clinical Studies

### Regulatory Considerations

Clinical trials are required to demonstrate safety and efficacy for virtually all Class III medical devices. They must be conducted using devices whose design and manufacture under Quality Systems (QS) are those of the product to be approved and that are used for the indications to be represented eventually in its labeling. This labeling includes all promotional materials and instructions to clinicians, engineers and patients. Clinical trials must be conducted according to strict protocols and record-keeping under Good Clinical Practices (GCP). Permission to conduct clinical trials in the US depends on filing applications for Investigational Device Exemption (IDE) by the FDA as well as obtaining local IRB approval for the identical protocol. The FDA requires that the outcome measures and the numbers of subjects will provide a valid and statistically relevant test of the proposed claims. The FDA and the IRB will also judge whether the likely benefits will outweigh the risks based on the sponsor’s analysis and preclinical *in vitro* and *in vivo* test data, and that the subjects will be adequately informed of and accept all risks.

For truly novel devices and clinical applications, suitable patient selection criteria, outcome measures and research protocols may not be apparent initially. The FDA encourages applicants to seek their advice about the possibility of an Early Feasibility trial, involving a few patients to validate specific questions relevant to the development of the product before the device design is fully fixed or before manufacturing methods are tightly controlled. If asked, the FDA may consider clinical results from prior academic research not under GCP. More traditionally, however, the clinical trial phase is characterized by two stages of clinical studies, a small pilot or feasibility phase using the near-final design produced under modified QS, and a much larger pivotal trial to collect statistically meaningful data. Feasibility studies are often conducted with grant funding at the academic institution of the founders; pivotal trials are usually conducted at multiple clinical centers to assure broader usability of the system. Devices can be sold at cost for such studies but that is usually not feasible in the absence of insurance or research grant coverage at this stage. The number of subjects in the pivotal trial must be justified by a statistical power analysis, which takes into consideration the size of the anticipated improvements in the outcome measures and the likely standard deviation of such results (Sakpal, 2010). At each stage, applicants are encouraged to request a presubmission meeting with the FDA to discuss their draft plans to demonstrate safety and efficacy. Note that the FDA will respond to such plans but will not propose a plan itself.

### Reimbursement Considerations

Reimbursement decisions of the US Centers for Medicare & Medicaid Services (CMS) are based on the criteria of “reasonable and necessary” (https://www.cms.gov/newsroom/fact-sheets/medicare-coverage-innovative-technology-cms-3372-f), as opposed to the criteria of “safe and effective” used for FDA regulatory approval. The various, highly balkanized health insurance providers in the US tend to make their own decisions, often on business and political grounds, for expensive new treatments for visibly disabled patients. Devices similar to those already reimbursed may be able to use existing coverage decisions, billing codes and payment amounts; novel sensorimotor prosthetics must seek approval through compelling, evidence-based arguments. A treatment may be deemed experimental and thus excluded from coverage long after it is FDA approved, especially when the treatment targets uncommon disorders. From a business perspective, insurers tend to regard treatments that add costs for continuing management and potential complications as undesirable, regardless of their ability to enhance quality of life. Treatments that produce a net savings in the insurance obligation by reducing the need for covered personal care enable a more powerful argument for reimbursement. Demonstrating such benefits usually requires longer data collection than for PMA, so it is useful to design the IDE trials with reimbursement requirements in mind (Ciani et al., 2017).

Failure to identify and pursue a well-established reimbursement path can substantially delay revenue generation. The necessary submissions to obtain a new coverage decision or code can take months or years to approve, and negotiations on price can be challenging given the many players. Reimbursement methods and decisions are not harmonized from one country to another, so the process must be understood for every jurisdiction in which the product is sold.

### Intellectual Property Considerations

Clinical studies are likely to produce results that the sponsors and participants will want to publish. These may well include inventive improvements to the technology or its methods of application that should be secured before they are made public. As a general rule, anyone who participates in discussions that result in claims must be included as an inventor. Some of those participants will likely be employees of the clinical study sites rather than the sponsoring company, so intellectual property rights need to be negotiated as part of the contract for these studies.

Decisions regarding international coverage of the foundational patents usually occur during the clinical studies phase. The trend now is to follow any provisional applications with Patent Cooperation Treaty (PCT) patent applications rather than going straight to US and other national utility patents (Lapenne, 2010). This strategy allows for various, substantial delays before formal applications must be filed in each national jurisdiction for which protection is desired. The expenses for translation, filing and prosecution differ greatly among nations. The PCT process includes an informal examination that may identify relevant prior art and help to shape the claims that will be filed and examined nationally. It should also be remembered that both PCT and US patent applications are published 18 months after the priority date, which will be the filing date of any referenced provisional application. This early publication exposes companies that may be operating in “stealth mode,” so it can be useful to identify potential competitors long before they appear in the marketplace. Similarly, clinical trials at US sites or under FDA IDE anywhere must be listed in www.ClinicalTrials.gov operated by the US Library of Congress.

### Funding Mechanisms

**FIGURE 3:**
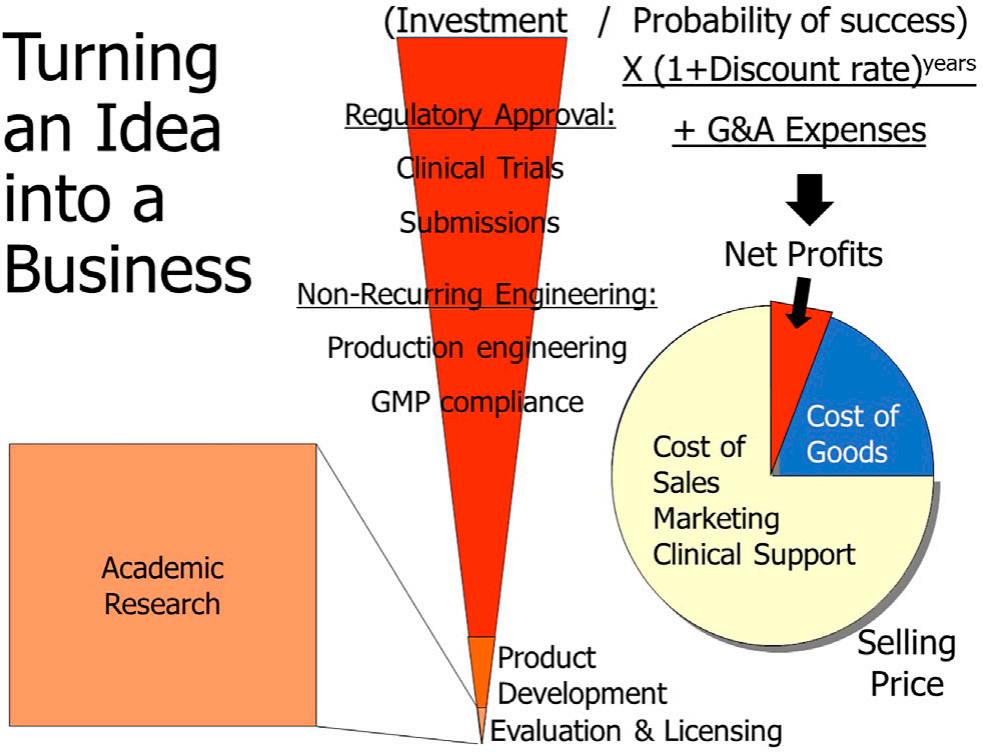
From the business perspective, research grants for academic research represent sunk costs that generate value only to the extent that they provide feasibility data for evaluation and licenses of intellectual property, both of which represent new costs to the company. Reengineering into a saleable product is usually necessary, but total investment (central red wedge) is eventually dominated by quality and regulatory requirements, particularly clinical trials. Return on this investment must come from net profits after cost of goods and costs of sales, marketing and clinical support, which usually dominate the selling price of medical products. The anticipated returns on an investment at any particular stage must take into account the probability of success at that point, the cost of money over the time before such earnings (discount rate), and the general and administrative (G&A) expenses to set up and run the business.

It is expensive to undertake controlled manufacturing and intensive clinical trials for devices that will be implanted in small numbers of clinical trial participants. Substantial outside investment (Series A, typically $5–10 M) is necessary and will usually be tied to milestones such as successful animal tests, IDE approval, and promising results from a clinical feasibility study. If the required pivotal trial will be expensive, a Series B capital raise (typically $10–30 M) will be tied to results from the feasibility study. This is the first of what will ultimately be a series of investments from private sources that thereby acquire partial ownership of the company and expect to be repaid from their share of profits from future sales (Figure 3). At each stage the dilution of ownership depends on the amounts of capital needed, the probability of success going forward and the valuation of the work and assets to date (Girling et al., 2010). From an investment perspective, the effort to date has a value based on a risk-adjusted projection of anticipated cash flow (net profits) less whatever investment will be required to achieve such revenue, a concept known as net present value (NPV) (De Visser et al., 2020). If the difference is positive, the investment is worthwhile financially.

Because of the high risk of any individual investment, venture capital funds will often pool their resources to fund many such projects in parallel, so the few successes must earn enough to pay for the many failures. The minimal return on investment depends on the probability of success at that stage, which is generally less than 10% for series A investments after demonstrating basic safety in animals but before clinical trials. For example, an investment of $10 M (covering the items in the red wedge in Figure 3) implies an expected (risk-adjusted) return of more than $100 M in profits from sales, plus adjustments for the cost of money between investment and exit. Net profits are typically about 10% of revenue from sales of product (https://guidingmetrics.com/content/medical-device-industrys-9-most-critical-metrics; https://www.statista.com/statistics/510318/gross-margin-and-net-margin-of-top-medical-device-companies) after accounting for the cost to produce each system and the costs associated with marketing, distribution, training and support (which tend to be the largest expenses for complex medical systems). This implies a market in excess of $1 B. If the product sells for $100 K, a market of at least 10,000 patients will be needed to break even. Series B and C funding will require further payback for what are then lower risk but probably larger investments. The share of equity in the company that is thus purchased by the various investors will usually be large and will usually entitle the investors to install whatever management they believe will maximize their return on investment. Inventors often fail to understand the business constraints that will require high prices and large sales volumes to ensure a viable commercial enterprise.

## Stage 3: PreMarket Approval

### Regulatory Considerations

The duration of the PMA review depends on the complexity of the product, the quality and integrity of the PMA submission and its underlying data, and political perspectives and personal idiosyncrasies at the FDA. Under the Medical Device User Fee Amendments III of 2012, PMAs require a submission fee of $365,657 as of 2021 ($91,414 for small businesses, first filing free). In return, the FDA commits to a variety of timely performance goals, including decisions within 320 days for PMAs that go to an Advisory Committee (https://www.fda.gov/media/83244/download). As of 2019, the FDA was meeting that goal fairly consistently (https://www.fda.gov/media/139848/download). But complex submissions often involve numerous interactions before they are accepted for review and after the FDA requests additional information. During the review period, each request can “stop the clock” until answers are received and thus add weeks or months to the review time.

### Reimbursement Considerations

During this stage, the company is likely to be continuing or extending clinical trials to gather the long-term cost/benefit data required for insurance coverage. Such activities are also useful in anticipation of marketing the approved product to the clinicians involved in such studies. These trials are expensive and generate no revenue. Much effort is currently directed at ways to collect data for reimbursement purposes from “real-world” sources, such as insurance databases, patient and disease registries or even social media, to buttress arguments for both regulatory and reimbursement decisions (Fleurence and Shuren, 2019; Pulini et al., 2021).

### Funding Mechanisms

During this stage, the company is expensive to run; it is operating at a high capital “burn rate” for an uncertain period. It must employ regulatory and engineering staff to respond to FDA queries and to recruit and train production and sales personnel in anticipation of market approval, but it has no revenue from sales of product. Clinical data will have reduced most of the existential risks about the clinical viability of the product but the delay until positive cash flow remains uncertain. These considerations determine the terms of further private equity capital raises (e.g. Series C in Figure 1) or perhaps acquisition by a larger company that already has resources for higher-volume manufacturing and international marketing, sales and clinical support.

## Stage 4: Postmarket Business

### Regulatory Considerations

Premarket approval by the FDA is not the end of regulatory requirements. Medical device manufacturers must operate under Quality Systems (QS) procedures that include monitoring of suppliers, acceptance testing of components, and corrective and preventive actions (CAPA) for deviations at any point in the supply, assembly and testing chain. Approvals of Class III devices often come with substantial requirements for post-market surveillance, in which long term data about performance, reliability and adverse events are reported regularly to the FDA (World Health Organization, 2020). The company will undergo regularly scheduled audits and inspections of its QS documentation and manufacturing facilities and records. Complaints can trigger unannounced inspections at any time.

Unlike drugs, approved medical devices often undergo substantial changes over time to add features, improve manufacturability or reliability or substitute for obsolete components. Such changes have been associated with adverse events (Zheng et al., 2017). Each change requires some type of notification or supplemental application to regulatory authorities, depending on the nature and risks of the change.

Medical products often start with narrow indications and claims based on the scope of their initial clinical trials and perhaps the limitations of a Humanitarian Use Designation. The real return on investment will come from extending the market beyond these tiny groups of patients. Physicians are allowed individually to prescribe and employ medical devices in ways that go beyond the strictly regulated labeling mandated by regulatory approvals, but companies must not advertise or promote such “off-label” use (Stafford, 2012). Such usage will often generate journal articles and provide the impetus to seek expanded regulatory approval, but such data will not be regarded as pivotal in regulatory applications unless they are obtained under a new IDE.

### Reimbursement Considerations

Initial reimbursement approval in one jurisdiction (e.g. CMS in the US or one National Health Service in Europe) is promising but does not guarantee approval by other payors (e.g. BlueCross/BlueShield in the US or another national or provincial health service). Getting physicians to prescribe and implant a novel device may require the company to create a new reimbursement pathway not only for the device but also for the associated new medical procedures.

### Intellectual Property Considerations

US patents have a lifetime of 20 years from date of filing, often time-stamped by a provisional application filed over a decade earlier during academic development. After the patent expires, competitors can introduce competing products that take advantage of the expensive pioneering by the first mover. Because the long regulatory process essentially deprives the patent holder of the opportunity for return on investment, the 1984 Drug Price Competition and Patent Restoration Act (also known as the Hatch-Waxman Act and interpreted to include medical devices) allows for a patent term extension of up to 5 years (https://grr.com/publications/the-hatch-waxman-act-and-its-effect-on-the-term-of-a-u-s-patent/). This must be applied for within 60 days of notification of premarket approval. The continuing evolution of a medical device mentioned above under Regulatory Considerations will often include patentable improvements. Successful medical device companies usually continue to develop patent portfolios aggressively as a barrier to entry for competitors.

### Funding Mechanisms

Return on the substantial investment in a medical device generally requires that a company achieve economies of scale. Cost of goods goes down when components can be purchased in bulk and labor can be organized into efficient assembly lines. Cost of sales and support can also decrease after a novel therapy becomes familiar and widely accepted. Because the reimbursed price for medical devices is rarely renegotiated, this offers an opportunity to increase gross profits. Once expenses associated with overall growth of the company stabilize, net profits may improve return on investment considerably.

Even without patent protection, clinician loyalty to familiar and reliable products can be a substantial barrier to entry for competitors. Most medical devices have a market lifetime of decades, as opposed to the 1–2 years typical of fast-changing consumer products. The prospect of a long-term “cash cow” is what attracts investors willing to accept the large, long-term and risky investments involved in medical product development. However, the increasing tendency for hospitals rather than physicians to dictate the purchase of medical devices can lead to increased renegotiation pressures on companies (Mosessian, 2016). The trend in the US appears to be to motivate intermediaries such as accountable care organizations to achieve quality and efficiency through the profit motive by reducing their costs (Colla and Fisher, 2017).

## Conclusion

It will be apparent to the reader that most of the activities described above require expertise in fields that are outside the training of most founding scientists, engineers and clinicians. Nevertheless, the vision of such founders is usually what started the project and their continuing guidance is usually necessary for it to succeed. Because pioneering a new medical product is lengthy, expensive and risky, investors need to maximize profits. This may entail changes to products, markets or promotional materials that conflict with the vision of the founders. Transitions in management, vision and control are thus inevitable but often painful (Wasserman, 2008).

It is important for both the founders and the investors to understand the motivations of the principals. These can generally be classified as fame, fortune or power. Most academic founders have already invested their careers in obtaining the respect of their peers and patients; they desire a place in medical history. Most investors put money at risk in order to earn more money. Most CEOs have invested in the skills required to lead great enterprises. Problems tend to arise when parties become confused about their real goals and capabilities. All parties need to remember that they will achieve their individual goals if and only if the whole enterprise succeeds through the combined efforts of those with complementary skills and motivations.

## Abbreviations

CAPA, corrective and preventive action; CE mark, conformity with European standards; CDRH, center for devices and radiological health; EU, European union; FDA, US food and drug administration; GCP, good clinical practices; GLP, good laboratory practices; GMP, good manufacturing practices for drugs; HDE, humanitarian device exemption; HUD, humanitarian use designation; IDE, investigational device exemption; NPV, net present value; PCT, patent cooperation treaty; PMA, pre-market approval; QS, quality systems for medical devices; SBIR, small business innovative research; STTR, small business technology transfer research; TPP, target product profile; 510(k), section of the US food, drug and cosmetic act for class II devices.
